# Nondestructive estimation of potato yield using relative variables derived from multi-period LAI and hyperspectral data based on weighted growth stage

**DOI:** 10.1186/s13007-020-00693-3

**Published:** 2020-11-10

**Authors:** Shanjun Luo, Yingbin He, Qian Li, Weihua Jiao, Yaqiu Zhu, Xihai Zhao

**Affiliations:** 1 Institute of Agricultural Resources and Regional Planning, Chinese Academy of Agricultural Sciences, Beijing, 100081 China; 2 School of Remote Sensing and Information Engineering, Wuhan University, Wuhan, 430079 China; 3 School of Economics and Management, Tiangong University, Tianjin, 300387 China; 4 Center for Agricultural and Rural Economic Research, Shandong University of Finance and Economics, Jinan, 250014 China

**Keywords:** Yield estimation, Remote sensing, Potato, Relative variables, Slogistic model, Weighted growth stage

## Abstract

**Background:**

The accurate estimation of potato yield at regional scales is crucial for food security, precision agriculture, and agricultural sustainable development.

**Methods:**

In this study, we developed a new method using multi-period relative vegetation indices (rVIs) and relative leaf area index (rLAI) data to improve the accuracy of potato yield estimation based on the weighted growth stage. Two experiments of field and greenhouse (water and nitrogen fertilizer experiments) in 2018 were performed to obtain the spectra and LAI data of the whole growth stage of potato. Then the weighted growth stage was determined by three weighting methods (improved analytic hierarchy process method, IAHP; entropy weight method, EW; and optimal combination weighting method, OCW) and the Slogistic model. A comparison of the estimation performance of rVI-based and rLAI-based models with a single and weighted stage was completed.

**Results:**

The results showed that among the six test rVIs, the relative red edge chlorophyll index (rCI_red edge_) was the optimal index of the single-stage estimation models with the correlation with potato yield. The most suitable single stage for potato yield estimation was the tuber expansion stage. For weighted growth stage models, the OCW-LAI model was determined as the best one to accurately predict the potato yield with an adjusted R^2^ value of 0.8333, and the estimation error about 8%.

**Conclusion:**

This study emphasizes the importance of inconsistent contributions of multi-period or different types of data to the results when they are used together, and the weights need to be considered.

## Background

Potato (*Solanum tuberosum* L.), a mixed grain, forage, and vegetable crop [1], is the fourth most important crop in the world [2, 3]. Since the launch of the potato staple food strategy in 2015 in China, potato has become another major staple food crop after rice, wheat, and corn [4]. Timely forecasting potato yield data is a vital reference index for variety breeding determined by the combination of genes and growth environment [5]. The accurate prediction of potato yield, especially at the regional level, is of great significance for ensuring food security and promoting the sustainable development of agriculture, which is related to the formulation of major policies and guidelines of the national economy and people's livelihood.

The method of crop growth models (CGM), costly, time-consuming, and not always accurate, is often used in conventional yield estimation, which relies on a large amount of data collection [6, 7]. It is reported that there are approximately 32 types of CGM combining multiple data sources and methods to monitor the potato yield under conditions of water, nitrogen fertilizer, and CO_2_ atmospheric levels [8]. However, the difficulty of obtaining large amounts of input data is one of the major limitations of the widespread employment of models due to their complexity [9–11]. Furthermore, field investigation, another traditional method, is a destructive estimation way. Although the accuracy of the final results can be guaranteed by comprehensive surveys, it is undoubtedly a laborious and time-intensive work [12, 13].

Remote estimation of yield is an approach to establish the relationship between crop spectra and yield data [14]. Remote sensing (RS), an emerging technique, can be used to effectively obtain spectral data of vegetation canopy from space in a non-destructive manner, which carries much valuable information indicating the interaction between canopy and solar radiation such as vegetation absorption and scattering [15]. Vegetation canopy spectrum is closely related to crop growth, especially the visible range affected by pigment and the near-infrared (NIR) bands affected by cell tissue and canopy structure [16, 17]. Therefore, the vegetation index (VI) calculated by these bands has been widely used for the monitoring and estimation of vegetation characteristic parameters, such as leaf area index (LAI) [18], biomass [19], chlorophyll content [20], nitrogen content and carbon content [21], and achieved high accuracy. In addition, various VIs showed great differences when applied in diverse scenarios. For example, when the fractional vegetation cover (FVC) is over 50%, the ratio vegetation index (RVI) has a high sensitivity for vegetation [22]. The normalized difference vegetation index (NDVI) is commonly used to research the vegetation growth and distinguish vegetation from non-vegetation with eliminating most of the radiation errors, but it is prone to saturation [23]. Not only that, VIs also have many applications in the yield estimation of different crops on account of the sensitivity to plant photosynthesis. Gong et al. [24] found that NDVI is of great help to the prediction of rapeseed yield using unmanned aerial vehicle (UAV) imagery. Moreover, VI also contributes significantly to yield estimation for crops such as rice [25, 26], maize [27, 28], and wheat [29, 30]. The simulation results of crop characteristic parameters can be obtained by constructing the linear or nonlinear empirical relationship [31] or by machine learning methods [32] like support vector machine (SVM), random forest (RF), partial least squares (PLS) and artificial neural network (ANN) between VIs and these parameters. So far, the VI-based parameter statistics is the simplest and most widely studied estimation method, which has been extensively applied in crop growth monitoring [33]. And the crop growth status monitored by RS directly determines the final crop yield. Hence, remote estimation of crop yield based on VI exhibits good potential, especially in a large-scale domain of estimation scenarios [34].

LAI is one of the vital parameters of crop canopy structure that related to photosynthesis, respiration, and transpiration [35]. Peng et al. [36] proved that LAI can be applied to estimate yield in oilseed rape using UAV data with the estimation error below 15%. Liu et al. [37] calculated the canopy density (Chl) using LAI and then constructed the simple linear prediction model of rice yield with an R^2^ value of 0.81. Therefore, LAI can be determined for yield estimation.

Employing RS technique including UAV, satellite, and ground measurement, massive data of multiple time series can be obtained. However, there are still some issues worthy of our attention. The atmospheric environment, soil background, and solar radiation conditions all will change during the process of obtaining data for many times [38]. Actually, eliminating the interference caused by illumination, aerosol, and background environment among multi-period data is a prerequisite for accurate yield estimation. For example, some reference whiteboards can be used for radiometric correction of remotely sensed images, but it is still difficult to obtain absolutely accurate data [39]. Therefore, we try to use the method of relative variables by subtraction to reduce the differences of data caused by the external environment.

In our experiment, the whole-stage canopy spectra of potato field were remotely measured from ground platforms, which had the advantage of reflecting the field variations well. Meanwhile, the LAI data in the same period were obtained. With potato grown under different water and nitrogen fertilizer treatments, our objectives are (1) to determine the optimal VI for single-stage yield estimation of potato; (2) to determine the optimal single stage for potato yield prediction, and (3) to compare the performance of rVI-based and rLAI-based models using single-stage and weighted stage data, and determine the final potato yield prediction model.

## Methods

### Study area

**Fig. 1:**
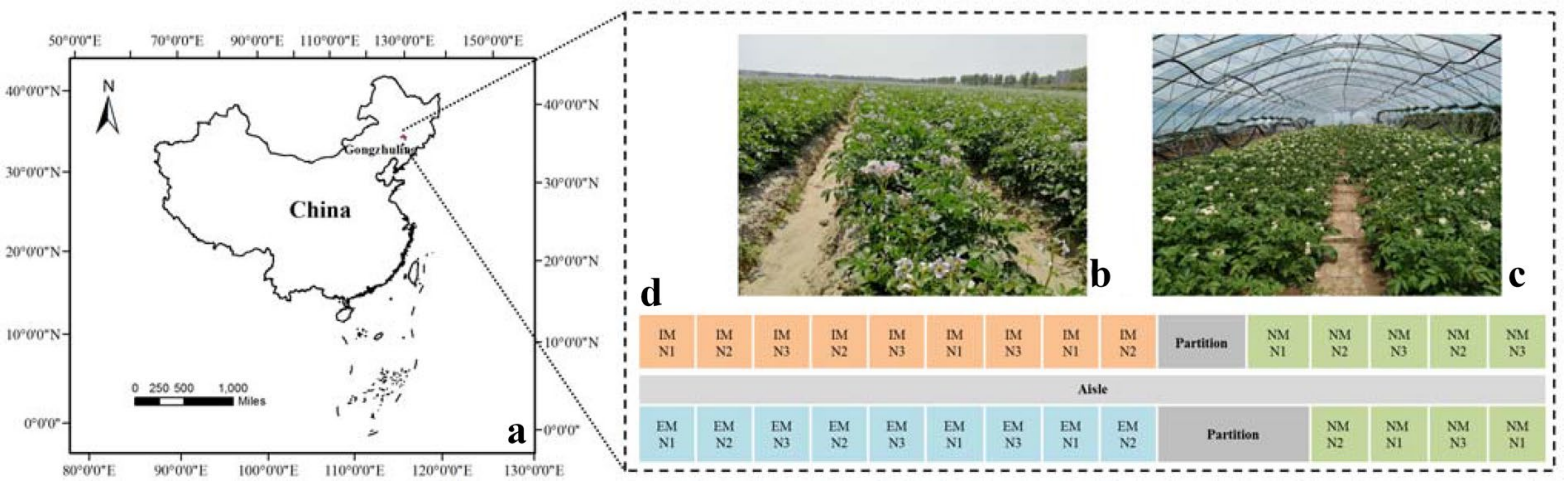
Study area location and plot distribution: **a** location of the study area; **b** field experiment; **c** greenhouse experiment; **d** water and nitrogen combination diagram of greenhouse

The experiment area (Fig. 1a) was located at the experimental base of Economic Plants Research Institute (43.45°N, 124.99°E), Jilin Academy of Agricultural Sciences, Gongzhuling City, Jilin Province, China. The greenhouse experiment (Fig. 1c) was conducted in May–September 2018. Shepody [40], a widely planted potato variety in Jilin Province, was selected as the experimental object. Potatoes were sown on May 2nd and harvested on September 10th including the whole growth stages. Through the combination of nitrogen fertilizer and water, 27 plots (Fig. 1d) including three nitrogen levels (N1: half of the normal nitrogen fertilizer, N2: normal nitrogen fertilizer, and N3: two times normal nitrogen fertilizer) and three water levels (EM: excessive moisture, NM: normal moisture, and IM: insufficient moisture) were set up. The water–nitrogen combination experiment was divided into 9 treatments, and each treatment was repeated 3 times randomly. To ensure that there is no water interference between the treatments, two partitions between IM and NM, and three partitions between EM and NM were set. The same potato variety was planted in the field experiment (Fig. 1b) to avoid the influence of sampling on the greenhouse experiment. The field experiment was used to study the change of dry weight with time to simulate the growth of potato, while the greenhouse experiment was applied to estimate potato yield by measuring hyperspectral and LAI.

The experimental area was located in the middle of the Songliao Plain, with a temperate continental monsoon climate, an average temperature from May to August of 18–20 °C, and abundant natural resources. It is a key commodity grain base in China and a demonstration area for potato cultivation.

### Data collection and multi-period data processing

The collection of data covered five key stages of potato growth: seeding stage (SS), tuber formation stage (TFS), tuber expansion stage (TES), starch accumulation stage (SAS), and harvest stage (HS). The field data, including LAI and hyperspectral, were collected for five times from SS (14 June), TFS (28 June), TES (23 July), SAS (9 August), to HS (27 August).

The SUNSCAN Canopy Analysis System (Delta-T Devices, Ltd., Burwell, Cambridge, UK) [41] was used to acquire the potato LAI data under conditions of windless and stable light. Since potatoes were planted following the ridges, our measurements were made 5 times parallel to the ridges and perpendicular to the ridges, respectively. Five different places were selected for measurement in each plot, and the mean values of 25 measurements in total were taken as canopy LAI values of the plot.

The USB 2000 spectrometer (Ocean Optics, Inc., Dunedin, Florida, United States) [42] was adopted to collect potato canopy hyperspectral under cloudless and windless conditions, with a spectral sampling interval of 0.46 nm. The spectral measurement was performed daily from 10:00 to 14:00 with the field-of-view angle of 25°, the probe vertically downward and about 1 m away from the top of the potato canopy. The observation was repeated five times for each plot, and the average value was regarded as the canopy spectral reflection. The reference whiteboard (chemical composition is BaSO_4_) was used for relative radiometric correction prior to measurement.

Dry weight measurements of potato plants (including stems, leaves, roots, flowers, etc.) were conducted by destructive sampling. In each growth stage, the sampling interval is 3–6 days. Ten points were randomly selected for each measurement. The collected plants were dried in the laboratory after drying in the field until their weights remained unchanged when weighing again. The average value was taken as the dry weight data of this measurement. In total, 17 times of sampling were taken on June 14 (SS), June 22, June 25, June 28, July 1 (TFS), July 9, July 13, July 16, July 19, July 23 (TES), July 31, August 3, August 6, August 9, August 16, August 21 (SAS), and August 27 (HS), respectively.

At HS, the potatoes in all plots were harvested manually. Then plot-level potatoes were weighed immediately.

For LAI and VI of multiple periods, the utilization of relative VI (rVI) and relative LAI (rLAI) is expected to reduce the limitation of uncertain information about background, light and atmospheric conditions at different growth stages. Firstly, plot-level rVI and rLAI were proposed under the premise of the hypothesis that solar radiation, atmospheric conditions, and field background were similar at each data acquisition. A standard plot can then be selected as a reference to help diminish the difference caused by time. In this study, rVI, rLAI, and relative yield were calculated based on a reference of an appropriate plot. The calculation of rVI, rLAI, and relative yield was carried out through the differences of VI, LAI, and yield between the study plot and reference plot (Eqs. 1–3). The method of eliminating the influence of external factors by subtraction can keep the correlation between original data unchanged.

1 $$rLAI = {LAI}_{\left(mea\right)} - {LAI}_{\left(Ref\right)}$$

where *rLAI* is the plot-level relative LAI, *LAI*_*(mea)*_ is the measured LAI of a study plot, *LAI*_*(Ref)*_ is the measured LAI of reference plot.

2 $$rVI = {VI}_{\left(mea\right)} - {VI}_{\left(Ref\right)}$$

where *rVI* is the plot-level relative VI, *VI*_*(mea)*_ is the plot-level VI calculated by measured spectra, *VI*_*(Ref)*_ is the VI calculated by measured spectra of reference plot.

3 $$relative\, yield = {yield}_{\left(mea\right)} - {yield}_{\left(Ref\right)}$$

where *yield*_*(mea)*_ is the measured yield of a study plot, *yield*_*(Ref)*_ is the measured yield of the reference plot.

### Vegetation index selection

Many scholars have determined that the optimal bands for studying the relationship between vegetation spectra and biophysical parameters lie in the visible and near-infrared ranges [43, 44]. According to this, VIs of NDVI, CI_red edge_, CI_green_, EVI2, NDRE, and MTCI (Table 1) calculated by the green (550 nm), red (670 nm), red edge (720 nm), and near-infrared (800 nm) bands were built. The reason why these six VIs were selected is that many scholars have achieved good results in relevant studies.

**Table 1:** Vegetation indices used in this study

Vegetation indices	Formula	References
Normalized Difference Vegetation Index (NDVI)	(R_800_ − R_670_)/(R_800_ + R_670_)	Rouse et al. [45]
Red edge Chlorophyll Index (CI_red edge_)	R_800_/R_720_ − 1	Gitelson et al. [46]
Green edge Chlorophyll Index (CI_green_)	R_800_/R_550_ − 1	Gitelson et al. [46]
Two-band Enhanced Vegetation Index (EVI2)	2.5(R_800_ − R_670_)/(1 + R_800_ + 2.4R_670_)	Jiang et al. [47]
Normalized Difference Red edge (NDRE)	(R_800_ − R_720_)/(R_800_ + R_720_)	Gitelson et al. [48]
MERIS Terrestrial Chlorophyll Index (MTCI)	(R_800_ − R_720_)/(R_720_ − R_670_)	Dash et al. [49]

### Algorithms for determining the weights of growth stages

#### Slogistic model

The curve expression of the Slogistic model is shown as Eq. (4). With the increase of independent variable, the value of the dependent variable increases slowly at first, but rapidly in a certain range later. When the independent variable reaches a certain limit, the growth of the dependent variable tends to be slow, and the whole curve shows a shape of flat "S". This equation is extensively used in epidemiology and agrometeorology [50].

4 $$y= \frac{a}{1+b{e}^{-kx}}$$

where *a* refers to the maximum value of the dependent variable, *b* and *k* are the characteristic parameters of the Slogistic curve equation.

The first-order and second-order partial derivatives of the independent variable of Eq. (4) were calculated to obtain Eqs. (5) and (6). According to the trend of curve change, the Slogistic model can be divided into three parts: the range of $[0 \sim [\mathrm{lnb}-\mathrm{ln}(2+\sqrt{3})]/\mathrm{k}]$ is the gradually increasing stage, $[[\mathrm{lnb}-\mathrm{ln}(2+\sqrt{3})]/\mathrm{k }\sim [\mathrm{lnb}+\mathrm{ln}(2+\sqrt{3})]/\mathrm{k}]$ is the rapidly increasing stage, and $[[\mathrm{lnb}+\mathrm{ln}(2+\sqrt{3})]/\mathrm{k }\sim \mathrm{ \infty }]$ is the slowly increasing stage. When the independent variable is lnb/k, the increasing speed of the dependent variable reaches the maximum value. The establishment of the model is helpful to judge the potato growth stages and determine their weights.

5 $$\frac{dy}{dx}= \frac{{abk}^{-kt}}{{(1+{bk}^{-kt})}^{2}}$$

6 $$\frac{{d}^{2}y}{d{x}^{2}}= \frac{{abk}^{-kt}({abk}^{-kt}-k)}{{(1+b{e}^{-kt})}^{3}}$$

#### Improved analytic hierarchy process

Analytic hierarchy process (AHP) is a system analysis method that combines qualitative and quantitative analysis, which was put forward by T.L. Saaty, a famous American operational research scientist in the early 1970s [51]. The judgment matrix of the traditional AHP adopted a nine-scale method (1–9). The subjective factors of experts play a leading role, which will lead to the deviation of the evaluation results. In addition, if the judgment matrix is not consistent in the consistency test, it will destroy the main function of the AHP’s scheme optimization and sorting, with a large amount of calculation and low accuracy. The improved analytic hierarchy process (IAHP) developed a new three-scale method (0–2), which made it easy for experts to make a comparison of the relative importance of the two factors, without the need for a consistency test. Moreover, IAHP can greatly reduce the number of iterations, improve the convergence speed, and meet the requirements of calculation accuracy [52]. The specific calculation steps are as follows:

1. Construction of comparison matrix *A*(*a*_*ij*_).

As shown in Eq. (7), according to the relative importance of potato growth stages, a comparison matrix *A*(*a*_*ij*_)_*5*×*5*_ was constructed.

7 $$A\left({a}_{ij}\right)= \left(\begin{array}{ccccc}1& 0& 0& 0& 0\\ 2& 1& 0& 0& 2\\ 2& 2& 1& 2& 2\\ 2& 2& 0& 1& 2\\ 2& 0& 0& 0& 1\end{array}\right)$$

where 0 indicates that the stage *i* is not as important as stage *j*; 1 indicates that the stage *i* is as important as stage *j*; 2 indicates that the stage *i* is more important than stage *j*.

2. Construction of judgment matrix *B*(*b*_*ij*_).

Firstly, the importance coefficients (${\mathrm{r}}_{j}= \sum_{i=1}^{5}{b}_{ij}$) of five potato growth stages were calculated, and then the judgment matrix *B*(*b*_*ij*_) was constructed as shown in Eq. (8):

8 $$B\left({b}_{ij}\right)= \left\{\begin{array}{l}\frac{{r}_{i}-{r}_{j}}{{r}_{max}-{r}_{min}} \times \left(k-1\right)+1 {r}_{i}\ge {r}_{j}\\ {\left[\frac{\left|{r}_{i}-{r}_{j}\right|}{{r}_{max}-{r}_{min}} \times \left(k-1\right)+1\right]}^{-1} {r}_{i}<{r}_{j}\end{array}\right.$$

where $${r}_{max}=\mathrm{max}\left\{{\mathrm{r}}_{j}\right\}$$, $${r}_{min}=\mathrm{min}\left\{{\mathrm{r}}_{j}\right\}$$, $$\mathrm{k}= {\mathrm{r}}_{max}/{\mathrm{r}}_{min}$$.

3. Calculation of transfer matrix C(c_ij_) and quasi-optimal uniform matrix C^*^ (c_ij_^*^).

The elements in transfer matrix C(c_ij_) and quasi-optimal uniform matrix C^*^ (c_ij_^*^) need to meet Eqs. (9) and (10).

9 $$C\left({c}_{ij}\right)= \frac{1}{5}\sum_{t=1}^{5}\left(\mathrm{lg}\frac{{b}_{it}}{{b}_{jt}}\right)$$

10 $${C}^{*}\left({{c}_{ij}}^{*}\right)= {10}^{{c}_{ij}}$$

4. Weight determination.

The maximum eigenvalue and the maximum eigenvector of the quasi-optimal matrix C^*^ were calculated, and the weight of each growth stage can be obtained after normalization.

#### Entropy weight method

The entropy weight method (EW) determines the index weight according to the variation degree of each index value. It is an objective weighting method, which has been widely used in the fields of economy, engineering, and finance [53]. The advantage of this method is that it can avoid the influence of human factors, but it ignores the importance of the index itself. Sometimes the weight of the index determined is far from the expected result, and the dimension of the evaluation index cannot be reduced [54]. The data matrix of *G*(*g*_*ij*_)_*5*×*5*_ was constructed based on the potato characteristic parameters of different plot-level in different stages, then the entropy value (*e*_*j*_) and the difference coefficient (*d*_*j*_) of each growth stage were calculated as shown in Eqs. (11) and (12).

11 $${e}_{j}= -\frac{1}{\mathrm{ln}n}\sum_{i=1}^{n}\left(\frac{{\mathrm{g}}_{ij}}{\sum_{i=1}^{n}{\mathrm{g}}_{ij}}\mathrm{ln}\frac{{\mathrm{g}}_{ij}}{\sum_{i=1}^{n}{\mathrm{g}}_{ij}}\right)$$

12 $${d}_{j}= \frac{1-{\mathrm{e}}_{j}}{5-\sum_{j=1}^{5}{\mathrm{e}}_{j}}$$

The weight *w*_*j*_ of the growth stage *j* can be obtained by normalizing the difference coefficient *d*_*j*_ as shown in Eq. (13).

13 $${w}_{j}= \frac{{\mathrm{d}}_{j}}{\sum_{j=1}^{5}{\mathrm{d}}_{j}} (0<{w}_{j}<1, \sum_{j=1}^{5}{w}_{j}=1)$$

#### Optimal combination weighting method

An optimal combination weighting method (OCW) was employed to solve the proportion of weights in the combined decision-making based on obtaining subjective and objective weights, then the decision weights considering both subjective will and objective existence were obtained [55]. To select a set of weights with the largest total distance (R) between the subjective weights and objective weights, the weight determined by the subjective weighting method was written as ${\mathrm{W}}_{1}=({\mathrm{w}}_{1}^{1}, {\mathrm{w}}_{2}^{2}, {\mathrm{w}}_{3}^{3}, {\mathrm{w}}_{4}^{4})$, the weight determined by the objective weighting method was written as ${\mathrm{W}}_{2}=({\mathrm{w}}_{1}^{2}, {\mathrm{w}}_{2}^{2}, {\mathrm{w}}_{3}^{2}, {\mathrm{w}}_{4}^{2})$, and the combined weight determined by OCW was written as $\mathrm{W}=({\mathrm{w}}_{1}, {\mathrm{w}}_{2}, {\mathrm{w}}_{3}, {\mathrm{w}}_{4})$. The optimal combination weight can be obtained by constructing the optimization model of Eq. (14) below.

14 $$\left\{\begin{array}{l}maxR= \sum_{m=1}^{2}(1 - \sqrt{\frac{1}{5}\sum_{j=1}^{5}{({w}_{j} - {w}_{j}^{m})}^{2}}) \\ \sum_{j=1}^{5}{w}_{j}=1\end{array}\right.$$

### Leave-one-out cross-validation

**Fig. 2:**
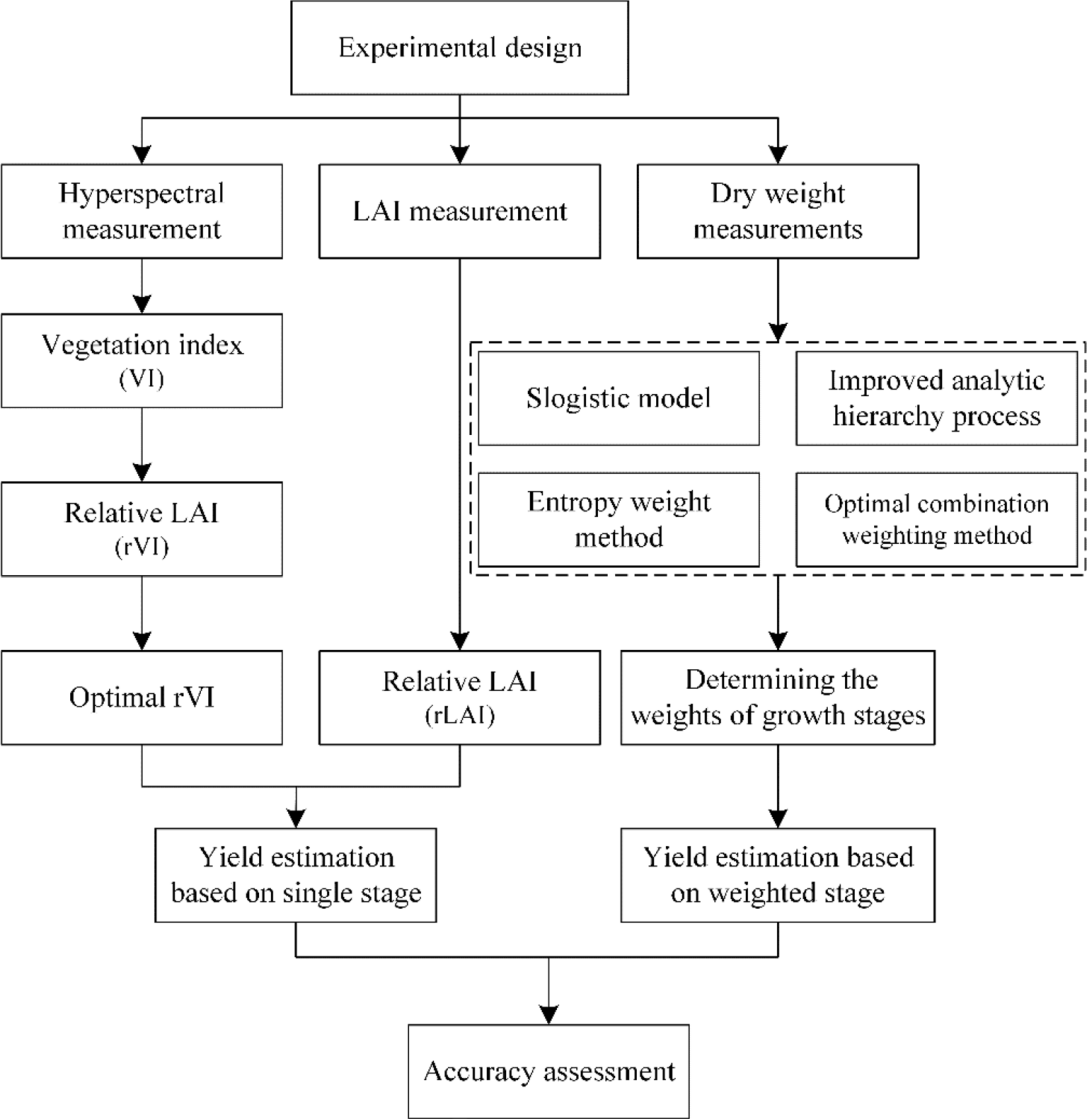
Technical flow chart in this study

The technical flow chart (Fig. 2) demonstrates the experimental methodology in this study, including experimental design, data collection, data processing, methods, and writing logic. The estimation and validation models of potato yield were established using leave-one-out cross-validation (LOOCV). This method is widely employed in model construction and validation to reduce the dependence on a single random part of the calibration and validation datasets [56]. Firstly, the original population samples were divided into K mutually exclusive sets (K = 26 in this study), of which K − 1 sets were used iteratively as training data for calibrating the coefficients (*Coef*_*i*_) of the algorithm, and then the remaining single sample was retained as the validation to obtain R^2^_i_ and the estimation error (*E(y*_*i*_*) *−* y*_*i*_). The whole training and validation process should be repeated K times until each sample participates in the validation process. After K iterations, the coefficients and precision of the final algorithm can be expressed as follows:

15 $$Coef = \frac{{\sum }_{i=1}^{K}{Coef}_{i}}{K}$$

16 $${R}^{2} = \frac{{\sum }_{i=1}^{K}{R}_{i}^{2}}{K}$$

17 $$RMSE = \sqrt{\frac{{\sum }_{i=1}^{K}{(E\left({y}_{i}\right)-{y}_{i})}^{2}}{K}}$$

where E(y) is the actual observed value, and *y* is the predicted value simulated by the model.

## Results

### Determination of the optimal rVI

**Fig. 3:**
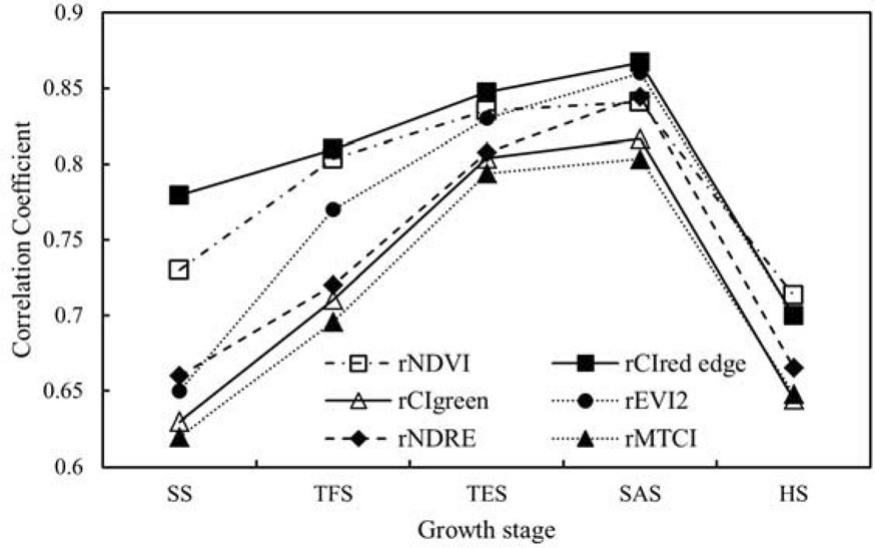
Comparison of correlation coefficients between different relative vegetation indices and yield

Each VI in this study was converted to rVI through the transformation of Eq. (2). The correlation coefficients between rVIs of different growth stages and relative yield are shown in Fig. 3. It can be seen that the correlation coefficients between each rVI and its corresponding relative yield showed an overall trend of increasing first (SS to SAS) and then decreasing (SAS to HS) during the whole growth stage. Correlated with relative yield, correlation coefficients of all selected rVIs in different stages exhibited consistent changes: reaching maximum values at SAS (with a correlation coefficient of 0.867 for rCI_red edge_, 0.860 for rEVI2, 0.845 for rNDRE, 0.841 for rNDVI, 0.817 for rCI_green,_ and 0.803 for rMTCI.) and showing smaller values at SS and HS. At each potato growth stage, there was the strongest correlation between rCI_red edge_ and relative yield. Therefore, it can be concluded that SAS is the most effective stage for potato yield estimating using VI, and rCI_red edge_ has the best performance. When using rVI to construct yield models, only rCI_red edge_ will be considered.

### Simulation of potato growth based on Slogistic model

**Fig. 4:**
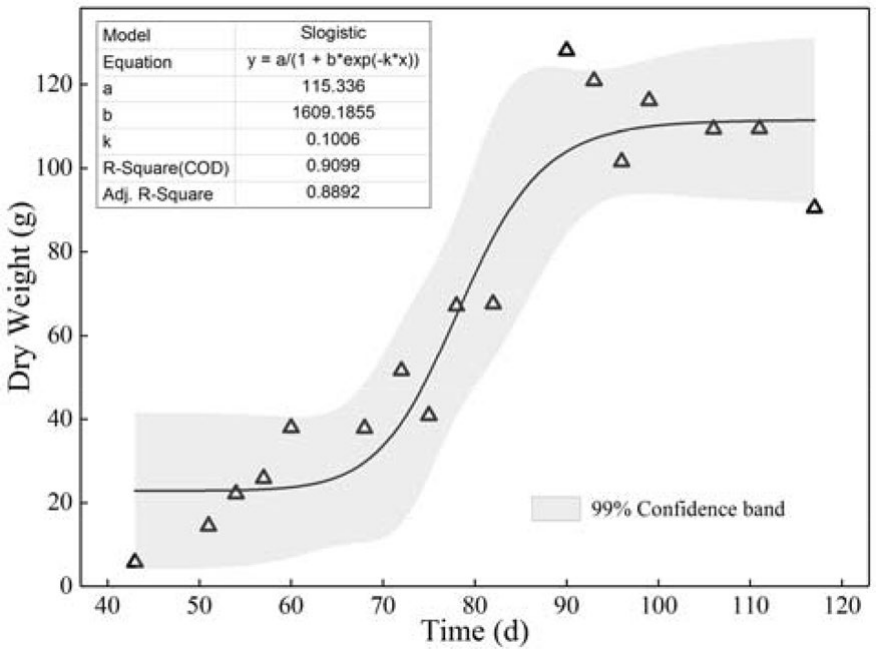
Variation of potato dry weight with time during the whole growth stage

As shown in Fig. 4, the dry weight data of the whole growth stage were used to construct the Slogistic model to characterize the growth process of potatoes. It can be found that the simulation accuracy is high, with the adjusted R^2^ close to 0.9. Generally speaking, the growth speed of potato is relatively slow in the early and late stages, and faster in the middle stage. Equations (5) and (6) were utilized to calculate the length of the growth stage, and the time nodes of the gradually increasing stage, the rapidly increasing stage, and the slowly increasing stage were 60th and 86th days, respectively. Based on these three stages, the five growth stages of this study can be obtained by increasing the seeding stage and harvest stage. The importance degree of each growth stage relative to yield can be sorted according to the growth rate of different stages. Combined with the actual planting experience, the final importance ranking was determined as TES > SAS > TFS > HS > SS. This result can provide a reference for the determination of the weights of different growth stages.

### Estimation of potato yield based on a single developmental stage

The new VI (rVI) and LAI(rLAI) datasets were compared with the relative yield data at five different developmental stages respectively. Adjust coefficient of determination (R^2^) and root mean square error (RMSE) of all estimation models of single-stage rLAI and rCI_red edge_ at each growth stage are shown in Table 2. At the same time, F-test was conducted on the whole regression models at 0.01 probability level, and the results were measured by P-value. Potato rLAI and rCI_red edge_ at TES closely related to the relative yield having the adjusted R^2^ above 0.7, much lower correlations were found at SS and HS. From the perspective of different stages, TES is the optimal stage when using rVI and rLAI to estimate potato yield, and the models' expressions are shown in Eqs. (18) and (19). In this stage, the prediction performance of VI is better than that of LAI (Adjusted R^2^ of 0.7415 vs. 0.7034, RMSE of 0.2671 vs. 0.2864).

**Table 2:** Potato yield estimation models for single stage

Growth stage	Regression equation	Adjusted R^2^	RMSE	Significance test
SS	y = 1.9752a_1_ − 0.1321	0.5912	0.3361	P < 0.001
	y = 1.6494b_1_ + 0.0935	0.4064	0.4043	P < 0.001
TFS	y = 0.4626a_2_ − 0.1644	0.6423	0.3145	P < 0.001
	y = 0.4223b_2_ − 0.0487	0.5661	0.3463	P < 0.001
TES	y = 0.4421a_3_ − 0.1823	0.7415	0.2671	P < 0.001
	y = 0.4305b_3_ − 0.1747	0.7034	0.2864	P < 0.001
SAS	y = 0.3436a_4_ + 0.0572	0.7063	0.2844	P < 0.001
	y = 0.3494b_4_ + 0.0593	0.6835	0.2956	P < 0.001
HS	y = 0.3269a_5_ + 0.0961	0.4692	0.3835	P < 0.001
	y = 0.3984b_5_ − 0.0706	0.5174	0.3654	P < 0.001

a1, a2, a3, a4, a5 are plot-level rVI (CI_red edge_ in this study) at SS, TFS, TES, SAS, and HS, respectively

b1, b2, b3, b4, b5 are plot-level rLAI at SS, TFS, TES, SAS, and HS, respectively

*y* is the relative yield

18 $${yield}_{(VI)} = {0.4421 \times r{CI}_{red\,edge}}_{[TES]} - {0.1823 +\mathrm{yield}}_{\left(Ref\right)}$$

where yield_(VI)_ is the estimated potato yield using single-stage rVI (rCI_red edge_ at TES in this study).

19 $${yield}_{(LAI)} = {0.4305 \times rLAI}_{[TES]} - {0.1747 +\mathrm{ yield}}_{\left(Ref\right)}$$

where yield_(LAI)_ is the estimated potato yield using single-stage rLAI.

### Estimation of potato yield based on weighted growth stage

Three weight calculation methods (subjectivity, objectivity, and their combined form) were used to determine the weights of potato growth stages (Table 3). The results showed that the weights of each growth stage determined by EW were very close. The weights determined by IAHP and OCW were the largest at TES, followed at SAS and TFS, and the smallest at HS and SS. Based on the weighting results of the three methods (IAHP, EW, and OCW), the rVI and rLAI data of the potato's critical growth stages in the study area were calculated (ie, the weighted rCI_red edge_ and rLAI), and then the linear regression models between the weighted relative variables and the relative potato yield were obtained. It can be found that the correlation between the potato yield and the weighted variables (rCI_red edge_ and rLAI) obtained by the three weighting methods was very significant (P < 0.001). For the three different weighting method models, the EW-based and OCW-based methods had the lowest and the highest model accuracy, respectively. But the results obtained by these three methods were significantly improved compared to the single-stage models. By comparing the fitting models of the two relative variables, the results obtained by the three weighting methods all showed that the weighted rLAI-based models had higher accuracy than the weighted rCI_red edge_-based models. The optimal estimation models of potato yield can be determined as Eqs. (20) and (21). As the final estimation models of potato yield were based on the relative yield model by adding the yield of the reference spot, their prediction ability remains unchanged (Table 3).

**Table 3:** Potato yield estimation models based on weighted growth stage

Weighting method	Vegetation parameter	SS	TFS	TES	SAS	HS	Regression model	Adjusted R^2^	RMSE	Significance test
IAHP	CI_red edge_	0.0329	0.1296	0.5101	0.2638	0.0636	y = 0.4484a − 0.1318	0.8092	0.2340	P < 0.001
	LAI						y = 0.4717b − 0.1778	0.8127	0.2318	P < 0.001
EW	CI_red edge_	0.1441	0.1556	0.2906	0.1633	0.2464	y = 0.5504a − 0.2357	0.7949	0.2426	P < 0.001
	LAI	0.1808	0.1701	0.2958	0.1679	0.1854	y = 0.6056b − 0.3255	0.7960	0.2419	P < 0.001
OCW	CI_red edge_	0.0663	0.1374	0.4442	0.2337	0.1185	y = 0.4775a − 0.1788	0.8225	0.2257	P < 0.001
	LAI	0.0773	0.1417	0.4458	0.2350	0.1002	y = 0.5169b − 0.2330	0.8333	0.2187	P < 0.001

*a* and *b* represent rCI_red edge_ and rLAI of weighted growth stage respectively. *y* is the relative yield of potato

20 $${yield}_{(VI)} = {0.4775 \times r{CI}_{red\,edge}}_{[weighted\,stage]} - {0.1788 +\mathrm{yield}}_{\left(Ref\right)}$$

where yield_(VI)_ is the estimated potato yield using rVI based on the weighted growth stage.

21 $${yield}_{(LAI)} = {0.5169 \times rLAI}_{[weighted\,stage]} - {0.2330 +\mathrm{ yield}}_{\left(Ref\right)}$$

where yield_(LAI)_ is the estimated yield using rLAI based on the weighted growth stage.

### Accuracy assessment using leave-one-out cross-validation

**Fig. 5:**
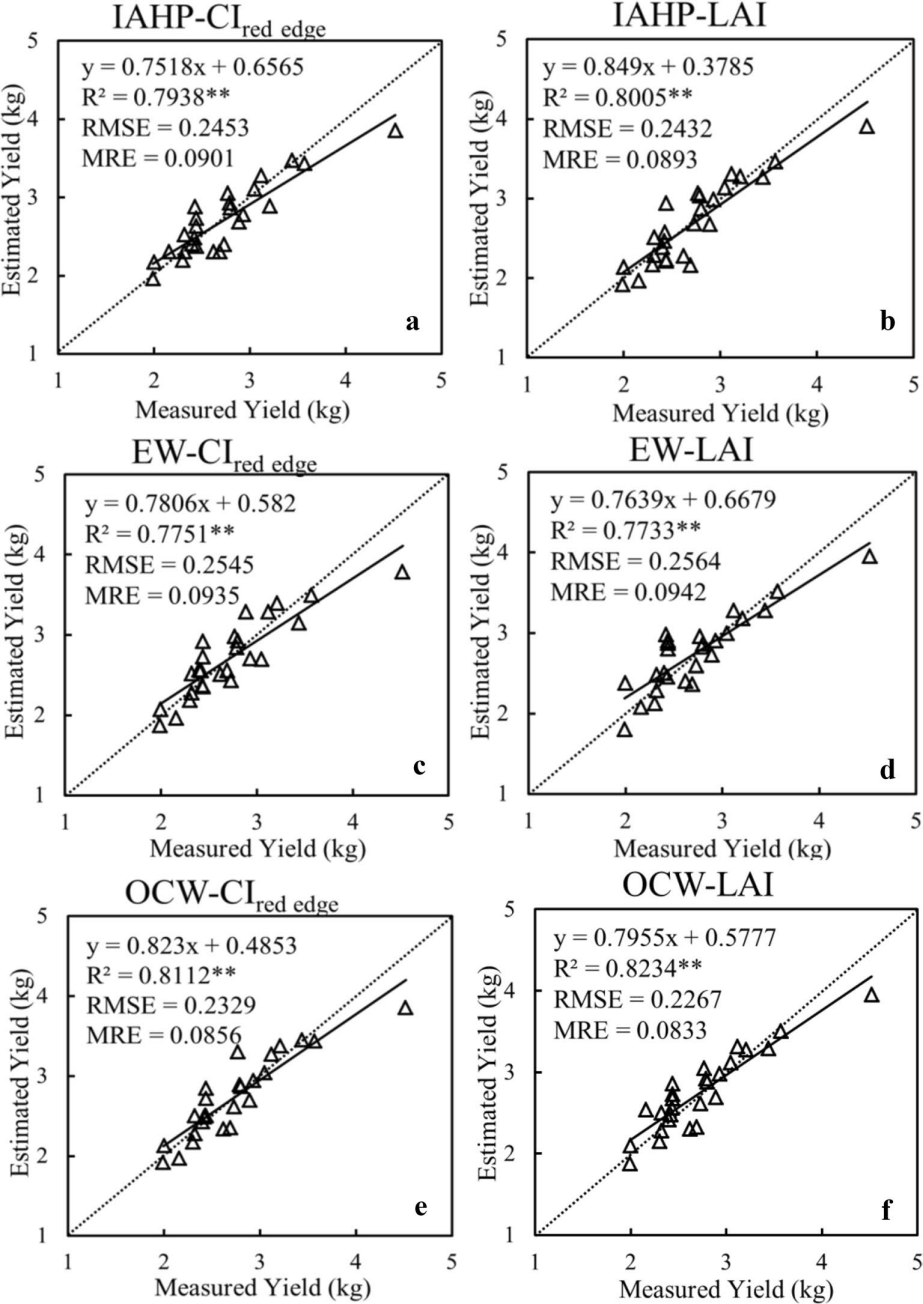
Validation models of potato yield estimation based on weighted growth stages and different variables: **a** IAHP-CI_red edge_; **b** IAHP-LAI; **c** EW-CI_red edge_; **d** EW-LAI; **e** OCW-CI_red edge_; **f** OCW-LAI

The leave-one-out cross-validation (LOOCV) method was utilized to obtain the potato yield validation models (Fig. 5). R^2^, RMSE, and mean relative error (MRE) were taken as evaluation indices. The results indicated that the accuracy of all models was acceptable (R^2^ > 0.75 and RMSE < 0.26). In general, models with high simulation accuracy also have high verification accuracy, with the minimum error less than 9%. Based on the combination models of three weighting methods and two different variables, the EW-based LAI model has the lowest accuracy, while the OCW-based LAI model has the highest accuracy (R^2^ = 0.8234, RMSE = 0.2267, MRE = 0.0833), explaining 82% of the variability. Therefore, combining the estimation and the verification models, the LAI model based on the OCW method to determine the weights of different growth stages is the optimal model for potato yield estimation.

## Discussion

For potato yield estimation, most scholars used to employ some crop growth models derived from general crop growth models or from gramineous (rice, wheat, corn, etc.) crop growth models [57]. Based on the principle and structure of the original model, the corresponding parameters were modified to conform to the growth characteristics of potato, and the growth process of potato was simulated, so as to output the physiological characteristic parameters and yield data and realize the model simulation function. Quiroz et al. [3] proposed that the incorporation of remotely sensed data in crop growth models with different temporal resolutions and levels of complexity could help to improve the yield estimation in potato. Moreover, it was identified that LAI at the initiation of stem elongation stage was closely related to yield, thus the remote estimation of LAI at this stage could be used to indicate the yield in oilseed rape [36]. Sharma et al. [58] tested Trimble GreenSeeker^®^ (TGS) and Holland Scientific Crop Circle™ ACS-430 (HCCACS-430) wavebands to predict potato yield using LAI and NDVI with R^2^ reaching 0.7. These studies indicate that both remote sensing and LAI data have potential for yield prediction. Therefore, spectra and LAI data were selected in this paper to estimate potato yield.

Six VIs of NDVI, CI_red edge_, CI_green_, EVI2, NDRE, and MTCI were utilized to non-destructively estimate potato yield in this study (Table 1) and CI_red edge_ showed the most excellent performance in the correlation with potato yield (Fig. 3). Gong et al. [24] also proved that CI_red edge_ had a good effect on the estimation of rapeseed yield using UAV data. Ma et al. [43] pointed out that at the seeding and bolting stage, the CI_red edge_ exhibited good performance compared to the other VIs. These conclusions are consistent with the results of this study, proving the credibility of this study.

In this study, the concepts of rVI and rLAI were proposed to solve the problem that the data acquired in different stages would be affected by solar radiation, aerosol, and soil background. Under the assumption of constant external conditions, subtraction can effectively remove these interferences, so that multi-period data can be used in combination. Furthermore, this method has the advantage of not changing the degree of aggregation and preserving the deviation of the original data. Wang et al. [59] used division to construct several relative vegetation indices (ΔVI) to estimate rice yield with hyperspectral imagery. Although the influence of external conditions such as background can be eliminated to some extent, the problem of changing the aggregation degree of data is ignored, resulting in lower RMSE and larger R^2^.

The dry weight data of the whole growth period were used to fit the Slogistic model (Fig. 4) by analyzing the growth process of potato (the growth rate is slow in the early and late stages, and fast in the middle stage). According to the model, we can not only divide the different growth stages of potato but also provide the basis for determining the weights of each growth stage. There are few systematic and specific divisions of potato growth stages in the existing literature. The main reason is that potato tubers are buried in the soil, and the changes can not be observed directly by the eyes. Therefore, the joint utilization of potato multi-period data is subject to certain restrictions [60]. With a clear division of growth stages, more refined research can be carried out like crops such as rice [61] and wheat [62].

At present, there are many problems about the joint use of multiple-period or various kinds of data. To improve the accuracy of the research results, many scholars blindly used the data of multiple-period or diversified data directly. For example, Zhou et al. [63] predicted rice grain yield using multiple linear regression (MLR) with multi-temporal VIs derived from the multi-spectral and digital images to improve the estimation accuracy. Obviously, the contributions of different developmental stages to yield estimation are not consistent, so it can not be directly used for MLR, ignoring the weights of growth stages. Wang et al. [64] estimated LAI of paddy rice using MLR, partial least squares (PLS) regression, and least squares support vector machines (LS-SVM) regression with 15 optimal hyperspectral bands to product more accuracy. No research has shown that the contributions of these 15 bands to LAI estimation are the same, so these data can not be directly used together. Of course, if multiple data are obtained in the same period, it can be used directly in combination. For example, Duan et al. [35] predicted rice LAI using SVM regression with spectral features and the texture features to determine the texture feature effective. In this study, IAHP, EW, and OCW methods were employed to confirm the weights of different stages of potato. From the perspective of subjectivity, objectivity, and the combination of them, the most suitable method (OCW) was selected, which solved the problem of joint use of multi-period data. The weighting results (Table 3) of different potato growth stages determined by EW are relatively close, thus they cannot reflect the degree of impact of different growth stages on yield. The calculation results of IAHP and OCW are in accordance with the actual situation.

When the spectra and LAI data of a single stage were used to predict the potato yield, the estimation accuracy of each stage basically met (1) TES > SAS > TFS > HS > SS for the same variable, which is consistent with the ranking of weights determined by IAHP, EW, and OCW; (2) VI > LAI for the same stage (Table 2). At HS, the simulation accuracy of VI is lower than that of LAI (adjusted R^2^ of 0.4692 vs. 0.5174), and when using the variable of VI, the accuracy at HS is lower than at SS (adjusted R^2^ of 0.4692 vs. 0.5912). The reason for this result is probably that the withering of potato leaves at HS resulted in the change of canopy spectra and the decrease of yield prediction ability.

**Fig. 6:**
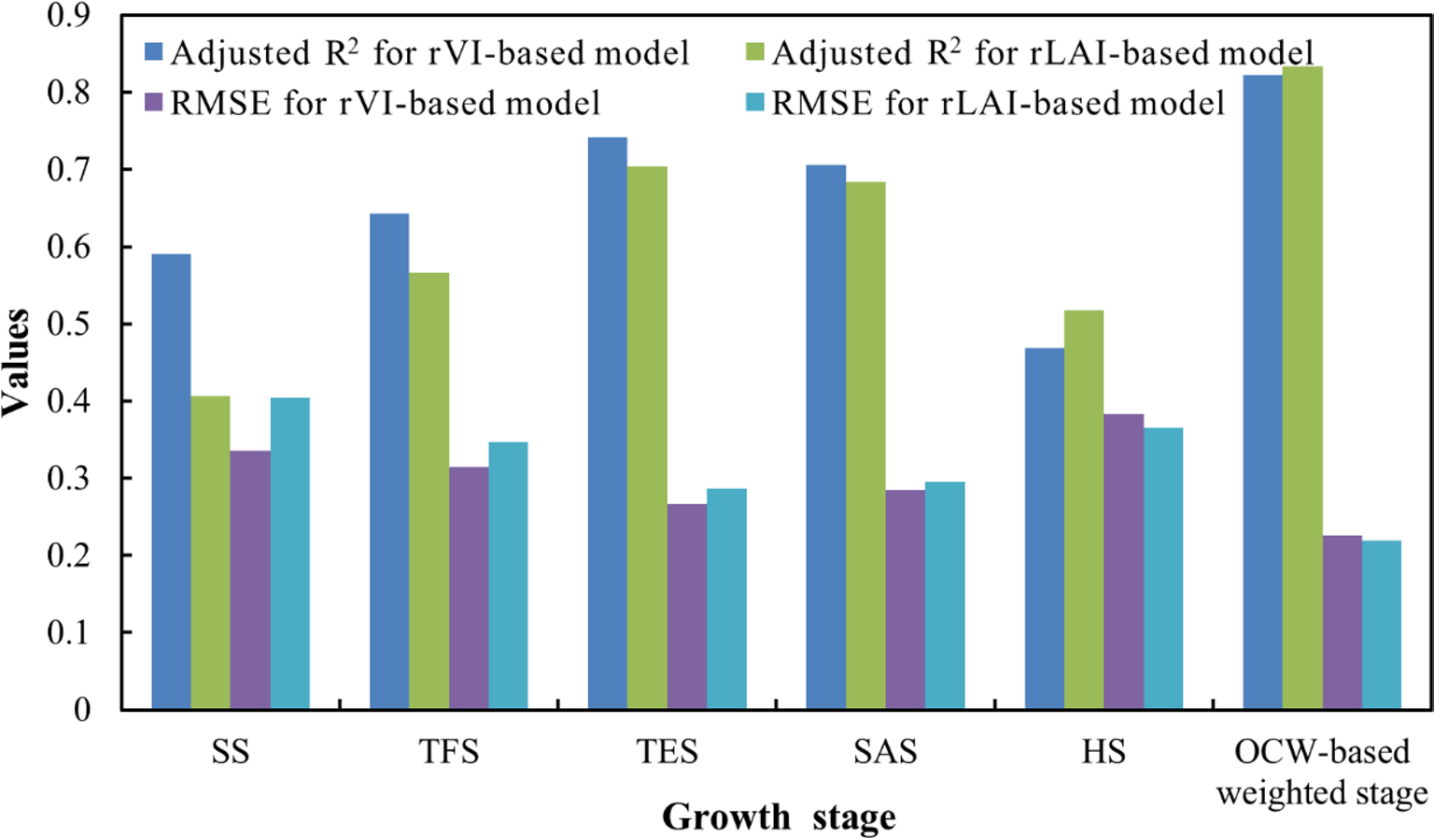
Adjusted R^2^ and RMSE of the different models

To compare the accuracy of yield estimation, the linear regression models were constructed based on plot-level weighted variables (rVI and rLAI) and relative yield (Table 3). The accuracy of different models was shown in Fig. 6. It can be found that OCW-based models have the highest accuracy. Unlike the single-stage results, in the OCW-based models, the accuracy of the rLAI model is higher than that of the rVI model. Because the appearance of saturation phenomenon in yield estimation using spectral index will limit the accuracy of models to some extent [25]. The LAI data is the three-dimensional (3D) information of the crop, and the limitations will be reduced.

To improve the suitability of the model, this experiment was set as water and fertilizer conditions, which can meet the current situation of water stress in potato planting areas in China and even the world [65]. Our future work will contain more data from different platforms for analysis, especially the UAV and satellite data because they can well express data at the spot-level. In addition, we will conduct experiments in more regions to verify the robustness of the models. And a new instrument of the LI-3100C table leaf area meter, (LI-COR Inc., LincoIn, Nebraska, USA) [66] will be used to avoid the impacts of the stems and flowers on the output of LAI, and more realistic LAI data will be obtained to improve and validate the accuracy of potato yield estimation by ground-measurement data.

## Conclusions

In this study, we developed a technique to improve the estimation of potato yield using weighted relative variables at plot-level derived from multi-period LAI and hyperspectral data. Plot-level relative vegetative index and LAI (rVI and rLAI) were proposed to eliminate the influence of external factors (solar radiation, aerosol, and soil background). The weights of different growth stages of potato were determined based on the Slogistic model and three weight calculation methods (IAHP, EW, and OCW). The linear regression was performed to estimate potato yield using single-stage and weighted multiple-stage variables respectively. The results indicated that rCI_red edge_ was the optimal index for the potato yield estimation among all the test rVIs. TES is most suitable for potato yield estimation using a single growth stage. When multi-period data were applied to estimate the potato yield, the accuracy was greatly improved. The estimation model of LAI using the OCW-based method combining subjectivity and objectivity (OCW-LAI) showed the best performance with the estimation error about 8%.

Although the idea of weighted developmental stage based on the Slogistic model and weighting calculation methods proposed in this study were tested in potato yield estimation, this work may offer a theoretical reference for other key parameters retrieving in crops that have an apparent division of growth stages. In future work, we will attempt to apply this technique to predict other growth parameters in potato and other crops.

## Data Availability

The remotely sensed and yield data used in this study is available upon the approval of Dr. Yingbin He from the Institute of Agricultural Resources and Regional Planning, Chinese Academy of Agricultural Sciences, China.
